# Supplementary material for: Health literacy assessment and healthcare access difficulties of Vietnamese migrants in Japan: A cross-sectional study
Source: PLoS One. 2026 Mar 20;21(3):e0344665. doi: 10.1371/journal.pone.0344665 (PMC13004346; doi:10.1371/journal.pone.0344665)
Supplement: Attachment — Submitted filename: PONE-D-25-55029_reviewer.pdf [file pone.0344665.s004.pdf]

PLOS ONE

Health literacy assessment and healthcare access difficulties of Vietnamese migrants in Japan: a cross-sectional study

--Manuscript Draft--

**Manuscript Number:**	PONE-D-25-55029
**Article Type:**	Research Article
**Full Title:**	Health literacy assessment and healthcare access difficulties of Vietnamese migrants in Japan: a cross-sectional study
**Short Title:**	Health literacy assessment and healthcare access difficulties of Vietnamese migrants in Japan
**Corresponding Author:**	Nobuo Kawazoe, PhD<br>Nagoya University of Commerce and Business Business School: Nagoya Shoka Daigaku Business School<br>Nisshin, Aichi JAPAN
**Keywords:**	Health Literacy; self-reported measurement; test-based measurement; Healthcare Access; Vietnamese migrants
**Abstract:**	While the number of Vietnamese migrant workers in Japan has been increasing, their health literacy has emerged as a key concern for ensuring equitable access to healthcare services. This study aimed to quantitatively assess health literacy among Vietnamese migrants in Japan and to examine their healthcare utilization and the difficulties they encounter. Convenience sampling was employed, with Vietnamese support organizations across Japan invited to disseminate a web-based survey via social media platforms. Using both self-reported and test-based health literacy tools, we measured health literacy levels in a sample of 137 Vietnamese migrants. We identified disparities by gender and topic area, particularly in mental health and knowledge of sexually transmitted infections. Our findings underscore the importance of culturally tailored health education and community-based interventions to support the health of this population. The study highlights the need for cultural and linguistically adapted educational materials to improve equitable access to healthcare. We also hope that our work contributes to the ongoing dialogue on migrant health and the development of inclusive public health strategies.
**Order of Authors:**	Takashi Tsubakita, PhD
	Nobuo Kawazoe, PhD
	Nobuyuki Matsuo, MA
**Additional Information:**	
**Question**	**Response**
**Financial Disclosure**<br><br>Enter a financial disclosure statement that describes the sources of funding for the work included in this submission. Review the submission guidelines for detailed requirements. View published research articles from *PLOS ONE* for specific examples.<br><br>This statement is required for submission and **will appear in the published article** if the submission is accepted. Please make	Yes

sure it is accurate.<br><br>**Funded studies**<br>Enter a statement with the following details:<br>• Initials of the authors who received each award<br>• Grant numbers awarded to each author<br>• The full name of each funder<br>• URL of each funder website<br>• Did the sponsors or funders play any role in the study design, data collection and analysis, decision to publish, or preparation of the manuscript?<br><br>Did you receive funding for this work?	
Please add funding details.<br>as follow-up to "**Financial Disclosure**<br><br>Enter a financial disclosure statement that describes the sources of funding for the work included in this submission. Review the submission guidelines for detailed requirements. View published research articles from *PLOS ONE* for specific examples.<br><br>This statement is required for submission and **will appear in the published article** if the submission is accepted. Please make sure it is accurate.<br><br>**Funded studies**<br>Enter a statement with the following details:<br>• Initials of the authors who received each award<br>• Grant numbers awarded to each author<br>• The full name of each funder<br>• URL of each funder website<br>• Did the sponsors or funders play any role in the study design, data collection and analysis, decision to publish, or preparation of the manuscript?<br><br>Did you receive funding for this work?"	TT.<br>This study was supported by a grant from the Daiko Foundation of Japan.
Please select the country of your main research funder (please select carefully	JAPAN - JP

as in some cases this is used in fee calculation).<br>as follow-up to "**Financial Disclosure**<br><br>Enter a financial disclosure statement that describes the sources of funding for the work included in this submission. Review the submission guidelines for detailed requirements. View published research articles from *PLOS ONE* for specific examples.<br><br>This statement is required for submission and **will appear in the published article** if the submission is accepted. Please make sure it is accurate.<br><br>**Funded studies**<br>Enter a statement with the following details:<br>• Initials of the authors who received each award<br>• Grant numbers awarded to each author<br>• The full name of each funder<br>• URL of each funder website<br>• Did the sponsors or funders play any role in the study design, data collection and analysis, decision to publish, or preparation of the manuscript?<br><br>Did you receive funding for this work?"	
**Competing Interests**<br><br>Use the instructions below to enter a competing interest statement for this submission. On behalf of all authors, disclose any competing interests that could be perceived to bias this work—acknowledging all financial support and any other relevant financial or non-financial competing interests.<br><br>This statement is required for submission and **will appear in the published article** if the submission is accepted. Please make sure it is accurate and that any funding sources listed in your Funding Information later in the submission form are also	The authors have declared that no competing interests exist.

declared in your Financial Disclosure statement.<br><br>View published research articles from *PLOS ONE* for specific examples.<br><br>**NO authors have competing interests**<br><br>Enter: *The authors have declared that no competing interests exist.*<br><br>**Authors with competing interests**<br><br>Enter competing interest details beginning with this statement:<br><br>*I have read the journal's policy and the authors of this manuscript have the following competing interests: [insert competing interests here]*<br><br>* typeset	
**Ethics Statement**<br><br>Enter an ethics statement for this submission. This statement is required if the study involved:<br><br>• Human participants<br>• Human specimens or tissue<br>• Vertebrate animals or cephalopods<br>• Vertebrate embryos or tissues<br>• Field research<br><br>Write "N/A" if the submission does not require an ethics statement.<br><br>General guidance is provided below. Consult the submission guidelines for detailed instructions. **Make sure that all information entered here is included in the Methods section of the manuscript.**	Ethical approval for this study was obtained from the Ethics Committee of Nagoya University of Commerce and Business, (Approval No. 23034). All participants were informed in writing that their participation was voluntary, that they could discontinue the survey at any time without any disadvantage, that their responses would be kept confidential, and that the consent to participate was considered to be given by the act of completing the questionnaire.

Powered by Editorial Manager® and ProduXion Manager® from Aries Systems Corporation

**Format for specific study types**<br><br>**Human Subject Research (involving human participants and/or tissue)**<br>• Give the name of the institutional review board or ethics committee that approved the study<br>• Include the approval number and/or a statement indicating approval of this research<br>• Indicate the form of consent obtained (written/oral) or the reason that consent was not obtained (e.g. the data were analyzed anonymously)<br><br>**Animal Research (involving vertebrate animals, embryos or tissues)**<br>• Provide the name of the Institutional Animal Care and Use Committee (IACUC) or other relevant ethics board that reviewed the study protocol, and indicate whether they approved this research or granted a formal waiver of ethical approval<br>• Include an approval number if one was obtained<br>• If the study involved *non-human primates*, add *additional details* about animal welfare and steps taken to ameliorate suffering<br>• If anesthesia, euthanasia, or any kind of animal sacrifice is part of the study, include briefly which substances and/or methods were applied<br><br>**Field Research**<br><br>Include the following details if this study involves the collection of plant, animal, or other materials from a natural setting:<br>• Field permit number<br>• Name of the institution or relevant body that granted permission	
**Data Availability**<br><br>Authors are required to make all data underlying the findings described fully available, without restriction, and from the time of publication. PLOS allows rare exceptions to address legal and ethical concerns. See the PLOS Data Policy and FAQ for detailed information.	No - some restrictions will apply

Powered by Editorial Manager® and ProduXion Manager® from Aries Systems Corporation

A Data Availability Statement describing where the data can be found is required at submission. Your answers to this question constitute the Data Availability Statement and **will be published in the article**, if accepted.<br><br>**Important:** Stating 'data available on request from the author' is not sufficient. If your data are only available upon request, select 'No' for the first question and explain your exceptional situation in the text box.<br><br>Do the authors confirm that all data underlying the findings described in their manuscript are fully available without restriction?	
**Describe where the data may be found in full sentences. If you are copying our sample text, replace any instances of XXX with the appropriate details.**<br><br>• If the data are **held or will be held in a public repository**, include URLs, accession numbers or DOIs. If this information will only be available after acceptance, indicate this by ticking the box below. For example: *All XXX files are available from the XXX database (accession number(s) XXX, XXX.).*<br>• If the data are all contained **within the manuscript and/or Supporting Information files**, enter the following: *All relevant data are within the manuscript and its Supporting Information files.*<br>• If neither of these applies but you are able to provide **details of access elsewhere**, with or without limitations, please do so. For example:<br><br>*Data cannot be shared publicly because of [XXX]. Data are available from the XXX Institutional Data Access / Ethics Committee (contact via XXX) for researchers who meet the criteria for access to confidential data.*<br><br>*The data underlying the results presented in the study are available from (include the name of the third party*	The data sets are available upon request from: kawazoe@nucba.ac.jp

and contact information or URL).<br>• This text is appropriate if the data are owned by a third party and authors do not have permission to share the data.<br><br>* typeset	
Additional data availability information:	

Powered by Editorial Manager® and ProduXion Manager® from Aries Systems Corporation

Manuscript
Click here to access/download;Manuscript;Manuscript.docx

Health literacy assessment and healthcare access difficulties of Vietnamese migrants in Japan: a cross-sectional study

Takashi Tusbakita[1], Nobuo Kawazoe[2*], Nobuyuki Matsuo[2]

[1] Department of Management, Nagoya University of Commerce and Business, Nisshin, Aichi, Japan

[2] Department of Economics, Nagoya University of Commerce and Business, Nisshin, Aichi, Japan

Corresponding author

E-mail: kawazoe@nucba.ac.jp (NK)

Abstract

While the number of Vietnamese migrant workers in Japan has been increasing, their health literacy has emerged as a key concern for ensuring equitable access to healthcare services. This study aimed to quantitatively assess health literacy among Vietnamese migrants in Japan and to examine their healthcare utilization and the difficulties they encounter. Convenience sampling was employed, with Vietnamese support organizations across Japan invited to disseminate a web-based survey via social media platforms. Using both self-reported and test-based health literacy tools, we measured health literacy levels in a sample of 137 Vietnamese migrants. We identified disparities by gender and topic area, particularly in mental health and knowledge of sexually transmitted infections. Our findings underscore the importance of culturally tailored health education and community-based interventions to support the health of this population. The study highlights the need for cultural and linguistically adapted educational materials to improve equitable access to healthcare. We also hope that our work contributes to the ongoing dialogue on migrant health and the development of inclusive public health strategies.

Introduction

In 2022, the World Health Organization highlighted critical health disparities among the world's 280 million migrants, particularly low-skilled workers who face limited access to healthcare and an increased risk of mental health issues such as depression and post-traumatic stress disorder [1]. Sustainable Development Goal 3 of the UN's 2030 Agenda calls for universal health coverage, emphasizing equitable access to essential health services and affordable medicines [2]. Addressing migrant health requires comprehensive strategies, including enhancing physical and financial access, improving health education, ensuring culturally and linguistically appropriate care, and strengthening healthcare infrastructure [2].

As of 2023, Japan had 3.13 million foreign residents, representing a 10.9% increase from the previous year [3]. Vietnamese nationals constitute the second-largest foreign population in Japan. Despite this growth, language barriers and limited understanding of Japan's healthcare system continue to hinder access to healthcare services [2]. Although some support systems, such as multilingual materials, legal consultations, and migrant-friendly directories, have been introduced, navigating these systems still relies heavily on individuals or their employers [4]. Community-based support often remains limited to native-language networks, underscoring the need for broader access to health information, interpreter services, and culturally sensitive care

[5–6]. Local Vietnamese associations and public agencies have begun addressing these issues through health booklets and community outreach.

To promote healthier lives for migrants in Japan, it is essential to improve their health literacy, defined as the ability to understand health systems, access accurate information, and appropriately utilize healthcare [7–8]. Low levels of health literacy are consistently associated with poorer health outcomes, including reduced use of preventive care, higher hospitalization rates, and weaker self-management of chronic conditions [9]. To overcome these challenges, support from local communities is vital to help individuals navigate the health system and access the information they need [10]. Evidence suggests that, in rural areas of Vietnam, community dynamics play a substantial role in shaping individuals' health literacy [11]. In Vietnam, health literacy research often employs the 47-item European Health Literacy Questionnaire (HLS-EU-Q47), which is closely linked to education and socioeconomic status [12–13]. Additional studies have examined digital literacy, eye health literacy [14–15], mental health [16–17], knowledge of infectious diseases [18], and other related topics [19–20]. Outside Vietnam, studies indicate that language and cultural barriers continue to restrict access to health information, particularly for women and immigrants in countries such as Australia and the United States [21–22]. To the best of our knowledge, no prior study has specifically investigated health literacy among Vietnamese migrants in Japan.

Objectives

This study aimed to quantitatively assess health literacy among Vietnamese migrants in Japan and to examine their healthcare utilization as well as the difficulties they encounter.

Materials and methods

Participants

According to the Ministry of Health, Labor, and Welfare of Japan, there were 1.82 million foreign workers in Japan in 2022, of whom approximately 460,000 (25%) were Vietnamese. Based on prior studies, this survey focused on Vietnamese workers likely to face greater challenges in health literacy: those employed in wholesale and retail, food services and accommodation, construction, manufacturing, and agriculture-related sectors. The survey also included Vietnamese individuals living in Japan as students or family members. Convenience sampling was employed. Vietnamese support organizations across Japan were asked to disseminate a web-based survey through social media platforms. Participants were informed that participation was voluntary, that their data would remain confidential, and that they could withdraw from the study at any time. The participants recruitment period started on 28/07/2024 and ended on 16/01/2025. Only responses from individuals who provided informed consent were included in the analysis.

This study was approved by the Ethics Committee of Nagoya University of Commerce and Business (Approval No. 23034).

Questionnaire

The questionnaire included demographic and health-related items, such as age, sex, history of illness or injury while living in Japan, whether medical care was sought, difficulties encountered during care, preferences for Vietnamese versus Japanese medications, and perceived barriers to accessing services.

The 12-item Short-Form Health Literacy Questionnaire (HLS-SF12) [12], adapted from the HLS-EU-Q47 [23], is widely used in Asia. It assesses perceived difficulty with health-related tasks across three domains: healthcare (items 1–4), disease prevention (items 5–8), and health promotion (items 9–12). Items are rated on a four-point Likert scale ranging from “very difficult” (1 point) to “very easy” (4 points). The functional Health Literacy Scale (funHLS) [24] is a 25-item multiple-choice test measuring basic health knowledge regarding the body, illness, nutrition, and medical care. Each item presents a health-related term and asks respondents to select the most relevant option or choose “I don’t know.” Higher scores on this test indicate greater functional knowledge.

Data analysis

The number of respondents who reported illness or injury and their healthcare-seeking behaviors were recorded. Means and standard deviations were calculated for the three HLS-SF12 domains. For the funHLS, the correct answer rate for each item and the overall mean score were computed. All analyses were performed using Stata version 19.

Results

In total, 141 responses were received from Vietnamese migrants residing in Japan. Of these, 137 responses were included in the final analysis after excluding four with substantial missing data. Table 1 summarizes the demographic characteristics of the participants. The sample consisted of 50 men (36.5%) and 87 women (63.5%). The mean age was 29.1 years, with a median of 28.0 years. The largest age group was 25–29 years, followed by 20–24 and 30–34 years. In terms of education, 44.5% had graduated from university, 42.3% from high school, and 8.0% from graduate school. Only a few participants reported completing junior high school or other forms of education.

Table 1. Participants' demographic characteristics.

Variables		n (%)
Sex	All	137 (100%)
	Men	50 (36.5)
	Women	87 (63.5)
Age group, years	17-19	2 (1.5)
	20-24	30 (21.9)
	25-29	49 (35.8)
	30-34	30 (21.9)
	35-39	15 (10.9)
	40-44	11 (8.0)
	Mean 29.1	
	Median 28.0	
Education	Junior high school	5 (3.6)
	High school	58 (42.3)
	University	61 (44.5)
	Graduate school	11 (8.0)
	Others	2 (1.6)

Table 2 presents employment sectors, visa status, length of stay, and region of residence. Participants were engaged in diverse sectors, with machinery manufacturing (20.4%) and food manufacturing (19.7%) being the most common, together accounting for approximately 40% of the sample. Other sectors included construction, textile manufacturing, agriculture, and fisheries. A large proportion (43.1%) were categorized as "other," reflecting a variety of occupations.

Regarding visa status, most participants were either workers (43.1%) or "admitted trainees" (29.9%). The admitted trainee program refers to workers admitted under a government scheme allowing limited-term employment in Japan. Family members, and permanent residents,

and students, accounted for 10.9%, 8.0%, and 7.3%, respectively. Only one participant was classified as “other,” suggesting that most respondents held work-related visas.

In terms of length of stay, 36.5% had lived in Japan for 1–2 years, while 19.0%, 21.9%, and 17.5% had stayed for 3–4, 5–6, and 7 years or more, respectively. Only 5.1% had been in Japan for less than one year. The mean and median durations were 3.8 and 4.0 years, respectively.

Regarding region of residence, 48.9% lived in western Japan, followed by 25.5% in northern regions. Smaller proportions resided in central, eastern, and southern Japan.

Table 2. Participants’ living status.

Variables		n (%)
Employment sector	Machinery manufacturing	28 (20.4)
	Food manufacturing	27 (19.7)
	Construction	9 (6.6)
	Textile manufacturing	8 (5.8)
	Agriculture	4 (2.9)
	Fisheries	2 (1.5)
	Others	59 (43.1)
Visa status	Workers	59 (43.1)
	Admitted trainees	41 (29.9)
	Family members	15 (10.9)
	Permanent residents	11 (8.0)
	Students	10 (7.3)
	Others	1 (0.7)
Length of stay, years	< 1	7 (5.1)
	1-2	50 (36.5)
	3-4	26 (19.0)
	5-6	30 (21.9)
	7-	24 (17.5)
	Mean 3.8	
	Median 4.0	
Region of residence in Japan	North	35 (25.5)
	East	8 (5.8)
	Middle	25 (18.2)
	West	67 (48.9)
	South	2 (1.5)

Access to healthcare and related challenges

Among the 137 participants, 77 reported seeking medical care for illnesses or injuries since arriving in Japan. The main difficulties encountered included communication barriers (70.1%), long waiting times (59.7%), not knowing which hospital to visit (53.2%), and high fees (50.6%).

Additional issues included expensive medicines, limited understanding of the healthcare system, and uncertainty about where to purchase medicines (Table 3).

Table 3. Difficulties in doctor visits.

Difficulties[#]	n (%)
Total	77 (100%)
Communication	54 (70.1)
Long waiting time	46 (59.7)
Hospital selection	41 (53.2)
Expensive fee	39 (50.6)
Expensive medicines	33 (42.9)
Understanding healthcare system	25 (32.5)
Where to get medicines	23 (29.9)

[#]A participant answered multiple difficulties in this table.

In contrast, 27 participants reported experiencing serious illness or injury but did not seek medical care. Reasons included not knowing which hospital department to visit (70.4%), perceiving the condition as not severe enough (66.7%), difficulty communicating in Japanese (59.3%), and, to a lesser extent, lack of time, high costs, long waiting times, inconvenient transportation, and absence of nearby hospitals (Table 4).

Table 4. Reasons of avoiding visiting doctors when illness or injured.

Reasons#	n (%)
Total	27 (100%)
No knowledge about proper doctors	19 (70.4)
Seemed not so severe enough	18 (66.7)
Difficulties in Japanese communication	16 (59.3)
Lack of time	15 (55.6)
High costs	15 (55.6)
Long waiting time	15 (55.6)
Inconvenient transportation	8 (29.6)
Absence of nearby hospitals	5 (18.5)

#A participant answered multiple reasons in this table.

When asked about medication preferences, 79 participants reported using Japanese medicines. Reasons included trust in quality (89.9%), lower costs (88.6%), and, to a lesser extent, familiarity and the availability of medicines not found in Vietnam (Table 5).

Table 5. Reasons of taking Japanese medicines instead of Vietnamese ones.

Reasons#	n (%)
Total	79 (100%)
Trust in quality	71 (89.9)
Lower costs	70 (88.6)
Familiarity	37 (46.8)
Not found in Vietnam	29 (36.7)

#A participant answered multiple reasons in this table.

Self-reported measures

Table 6 shows participants' self-reported scores on the HLS-SF12 across three domains.

Table 6. Self-reported scores based on HLS-SF12.

Questions	All, n=137<br>Mean (SD)	Men, n=50<br>Mean (SD)	Women, n=87<br>Mean (SD)
**Health care**	9.12 (2.24)	9.04 (1.99)	9.16 (2.52)
Q1. Informaton on deseases	2.42 (0.73)	2.34 (0.66)	2.46 (0.77)
Q2. Understanding leaflets	2.33 (0.75)	2.32 (0.62)	2.33 (0.82)
Q3. Judgement the advantages of treatments	2.10 (0.70)	2.10 (0.68)	2.10 (0.72)
Q4. Amburance call	2.27 (0.81)	2.28 (0.76)	2.26 (0.84)
**Preventive medicine**	10.03 (2.48)	9.62 (2.36)	10.26 (2.52)
Q5. Finding informaton on deseases	2.35(0.76)	2.32 (0.68)	2.37 (0.81)
Q6. Understanding health screenings	2.52 (0.73)	2.44 (0.67)	2.56 (0.76)
Q7[#]. Judgement on the vaccination	2.49 (0.75)	2.30 (0.74)	2.60 (0.76)
Q8. Judgement on the treatment	2.67 (0.76)	2.56 (0.76)	2.74 (0.75)
**Health promotion**	10.72 (2.39)	10.76 (2.40)	10.70 (2.39)
Q9. Contact with health activities	2.80 (0.71)	2.84 (0.74)	2.77 (0.69)
Q10. Understanding on media information	2.80 (0.75)	2.84 (0.77)	2.78 (0.74)
Q11. Judgement on the healthy life style	2.79 (0.72)	2.76 (0.69)	2.80 (0.74)
Q12. Participating physical training activities	2.34 (0.81)	2.32 (0.82)	2.34 (0.80)

SD, Standard deviation

Men < Women (P value = 0.025)

The Q1-Q12 below are questions in the Table 6.

Q1. Do you find information on treatments of illnesses that concern you?

Q2. Do you understand the leaflets that come with your medicine?

Q3. Do you judge the advantages and disadvantages of different treatment options?

Q4. Do you call an ambulance in an emergency?

Q5. Do you find information on how to manage mental health problems like stress or depression?

Q6. Do you understand why you need health screenings (such as breast exam, blood sugar test, blood pressure)?

Q7. Do you judge which vaccinations you may need?

Q8. Do you decide how you can protect yourself from illness based on advice from family and friends?

Q9. Do you find out about activities (such as meditation, exercise, walking, Pilates etc.) that are good for your mental well-being?

Q10. Do you understand information in the media (such as Internet, newspaper, magazines) on how to get healthier?

Q11. Do you judge which everyday behavior (such as drinking and eating habits, exercise etc.) is related to your health?

Q12. Do you join a sports club or exercise class if you want to?

For the healthcare domain, the mean score (M) was 9.12, with no notable sex difference (men: 9.04; women: 9.16). Tasks such as "finding information on diseases" (M = 2.42) and

“calling an ambulance” (M = 2.27) were rated as easier, while “judging pros and cons of multiple treatment options” (M = 2.10) was considered most difficult. For the disease prevention domain**,** **t**he mean score was 10.03, with women scoring higher than men (10.26 vs. 9.62). Participants found “understanding the need for health checkups” (M = 2.52) and “judging the need for vaccinations” (M = 2.49) easier, while “finding mental health information” (M = 2.35) was more difficult. A significant sex difference was observed for “judging the need for vaccinations,” where women scored higher than men (2.60 vs. 2.30, p = 0.025).

For the health promotion domain, the mean score was 10.72, with no sex differences. Respondents rated “understanding health media information” (M = 2.80) and “judging healthy lifestyles” (M = 2.79) as easier. However, “participating in physical training activities” scored lower (M = 2.34), suggesting greater difficulty.

Test-based measures

The funHLS is a measurement tool of health literacy based on tests. Table 7 presents item-wise correct response rates for the 25 items.

Table 7. Test-based scores based on funHLS.

Question items	All, n=137 Ratio (SD)	Men, n=50 Ratio (SD)	Women, n=87 Ratio (SD)	p value
Q1. Caries	0.74 (0.44)	0.76 (0.43)	0.74 (0.44)	0.753
Q2. Prescription	0.61 (0.49)	0.48 (0.50)	0.68 (0.47)	0.022*
Q3. Generic drug	0.06 (0.24)	0.06 (0.24)	0.06 (0.23)	0.952
Q4. Measles	0.52 (0.50)	0.44 (0.50)	0.56 (0.50)	0.165
Q5. Sexually transmitted infections	0.23 (0.42)	0.14 (0.35)	0.28 (0.45)	0.067
Q6. Anemia	0.76 (0.43)	0.68 (0.47)	0.80 (0.40)	0.101
Q7. Potassium	0.56 (0.50)	0.54 (0.50)	0.57 (0.50)	0.693
Q8. AED	0.28 (0.45)	0.30 (0.46)	0.28 (0.45)	0.763
Q9. Fat	0.53 (0.50)	0.46 (0.50)	0.57 (0.50)	0.195
Q10. Depression	0.55 (0.50)	0.46 (0.50)	0.61 (0.49)	0.091
Q11. Amenorrhea	0.18 (0.38)	0.08 (0.27)	0.23 (0.42)	0.026*
Q12. Schizophrenia	0.65 (0.48)	0.58 (0.50)	0.69 (0.47)	0.195
Q13. Autism spectrum disorder	0.28 (0.45)	0.28 (0.45)	0.29 (0.46)	0.927
Q14. Revisit to clinic	0.55 (0.50)	0.60 (0.49)	0.52 (0.50)	0.349
Q15. Kidney	0.77 (0.42)	0.72 (0.45)	0.80 (0.40)	0.255
Q16. Pneumothorax	0.76 (0.43)	0.76 (0.43)	0.76 (0.43)	0.985
Q17. Medical treatment not coverd by insurance	0.46 (0.50)	0.36 (0.48)	0.52 (0.50)	0.075
Q18. Carbohydrates	0.57 (0.50)	0.54 (0.50)	0.59 (0.50)	0.599
Q19. BMI	0.50 (0.50)	0.48 (0.50)	0.52 (0.50)	0.675
Q20. Salt	0.68 (0.47)	0.62 (0.49)	0.71 (0.45)	0.264
Q21. Insurance card	0.82 (0.38)	0.84 (0.37)	0.82 (0.39)	0.723
Q22. Appendicitis	0.76 (0.43)	0.72 (0.45)	0.78 (0.42)	0.417
Q23. Vitamin C	0.63 (0.49)	0.54 (0.50)	0.68 (0.74)	0.107
Q24. Syphilis	0.83 (0.38)	0.78 (0.42)	0.86 (0.35)	0.216
Q25. Uterocervical cancer	0.31 (0.46)	0.26 (0.44)	0.33 (0.47)	0.370

SD, Standard deviation.

The p-value for sex-based comparison for each item is shown.

*p<0.05

Overall, correct response rates varied widely. High accuracy was observed for “syphilis” (83%), “health insurance card” (82%), “kidney” (77%), and “pneumothorax” (76%). Low rates were recorded for “generic drugs” (6%), “amenorrhea” (18%), “sexually transmitted infections” (23%), and “autism spectrum disorder” (28%).

Sex-based comparisons showed that women scored higher than men overall. Significant differences were found for “prescription” (68% vs. 48%, p = 0.022) and “amenorrhea” (23% vs. 8%, p = 0.026), where women outperformed men. None of the items showed significantly higher scores among men. By category, knowledge of infectious diseases and vaccinations varied: correct response rates were 52% for “measles,” 23% for “sexually transmitted infections,” and 83% for “syphilis,” reflecting relatively low awareness of sexually transmitted infections. Regarding mental and neurological health, correct response rates for “schizophrenia” (65%), “depression” (55%), and “autism spectrum disorder” (28%) showed that while some conditions were better understood, developmental disorders were less recognized.

Knowledge of nutrition and lifestyle was relatively stable, with moderate accuracy for “salt” (68%), “carbohydrates” (57%), and “BMI” (50%). However, responses for specific nutrients such as “potassium” (56%) varied, suggesting uneven understanding in this area.

Discussion

Access to healthcare and related challenges

Many Vietnamese migrants in Japan experienced illnesses or injuries and sought medical care. However, a notable proportion did not access medical services even when needed. The contributing factors included limited time due to work, high medical costs, and language barriers. Several participants reported confusion about which hospital to visit or how the healthcare system operates, indicating insufficient knowledge of Japan's medical infrastructure. Communication difficulties with medical staff were also common and may have hindered appropriate treatment. Long waiting times and high fees further discouraged access to care. Some participants avoided medical services because of difficulties navigating the system in Japanese or uncertainty about the appropriate department to visit. In some cases, geographic isolation and inadequate transportation also limited access.

Regarding medications, both Japanese and Vietnamese medicines were used. Japanese medicines were preferred for their reliability and affordability, while Vietnamese medicines were chosen due to familiarity and the availability of certain types not found in Japan. This reflects a continued demand for both.

In summary, although many Vietnamese migrants in Japan use healthcare services, access is often restricted by systemic, linguistic, and financial challenges. To improve health equity,

support through accessible medical information, stronger language services, and reduced financial burdens is necessary.

Health literacy from the perspective of task difficulty

In the healthcare domain of the HLS-SF12, no significant sex differences were observed. Participants found basic tasks such as finding information on illnesses or calling an ambulance relatively easy but had difficulty "judging the pros and cons of multiple treatment options." This suggests competence in basic health tasks but challenges with more complex decision-making, likely due to limited access to professional information or system-level knowledge. Language barriers and a lack of health-related education may also contribute to these challenges. In the disease prevention domain, women scored higher overall, particularly on "judging the need for vaccinations." This may reflect women's greater interest in preventive health or their increased exposure to related information. Conversely, low scores for "finding mental health information" suggest limited access to such resources or high barriers to seeking them.

In the health promotion domain, the mean score was 10.72, with no sex difference. Respondents found it relatively easy to understand health-related media information and wellness concepts, such as meditation or walking. However, lower scores for "participating in physical

training activities" suggest barriers to actual engagement, likely stemming from financial, time, or language constraints.

Test-based health literacy

Results from the funHLS varied depending on topic knowledge. Participants performed well on familiar items such as "insurance card," "syphilis," "kidney," and "pneumothorax," but poorly on "generic drugs," "amenorrhea," "sexually transmitted infections," and "autism spectrum disorder," which require more technical knowledge. Men scored particularly low on certain items compared with women, suggesting sex-based disparities in exposure or health-related knowledge.

Notably, awareness of sexually transmitted infections (STIs) was limited. It is possible that the Vietnamese term for STIs is not widely known or commonly used. While awareness of conditions such as measles and syphilis was higher, broader knowledge of infectious diseases was inconsistent. In mental health, participants showed moderate understanding of depression and schizophrenia but low recognition of autism spectrum disorder, suggesting insufficient awareness of developmental disorders and the need for targeted education [25].

Knowledge of basic nutrition topics such as salt, carbohydrates, and BMI was moderate, but responses regarding specific nutrients, such as potassium, were less consistent. These findings highlight gaps in access to detailed health information and uneven educational exposure.

Perspective on health literacy

Overall, the findings reveal uneven distribution of knowledge and skills, with challenges in complex decision-making and healthcare system navigation. Women generally scored higher than men, perhaps reflecting their greater tendency to seek health-related information. These results underscore the importance of actively supporting men's health literacy as well.

Multilevel approaches are required to improve health literacy among Vietnamese migrants in Japan. These include improving access to medical information, providing language support and targeted health education, and implementing strategies to reduce financial burdens. In addition to localized or workplace-based interventions, programs focusing on mental health and STIs are especially crucial. Sustained, culturally responsive efforts are essential to enhance the well-being and healthcare navigation skills of Vietnamese migrants in Japan.

Limitations

This study has several limitations. First, although participants were recruited from across Japan, the sample size was relatively small, and convenience sampling was used. Therefore, the findings may not be generalizable to the broader population of Vietnamese migrants in Japan. There may also be occupational biases. For example, 44.5% of respondents were university graduates,

whereas the estimated proportion of university graduates in Vietnam is about 20%. Thus, the sample was skewed toward individuals with higher educational attainment. Second, the study relied on self-reported data, which are subject to recall and social desirability biases. Participants may have over- or underestimated their health behaviors or knowledge. Finally, although this study considered linguistic and cultural barriers, it did not examine in detail how these factors specifically influenced health literacy. Future research should explore the roles of language proficiency, cultural adaptation, and prior health education more thoroughly. Despite these limitations, this study represents one of the first attempts to quantitatively assess health literacy among Vietnamese migrants in Japan and provides important insights to inform policy and intervention development.

Conclusion

This study highlights the importance of culturally and linguistically tailored educational materials in improving equitable access to healthcare. Effective measures may include interpretation services, navigation support, and targeted outreach through community and workplace channels. Strategies to better engage male migrants in health promotion are also recommended.

Acknowledgements

We would like to express our sincere gratitude to the Vietnamese residents in Japan who kindly participated in the survey. We also extend our appreciation to the members of Vietnamese associations who supported participant recruitment.

References

1. World Health Organization. World report on the health of refugees and migrants: summary World Health Organization; 2022.
2. United Nations. Transforming our world: the 2030 Agenda for Sustainable Development. [Cited 2025 October 1]. Available from: https://sdgs.un.org/2030agenda.
3. Immigration Service Agency of Japan. [Cited 2025 October 1]. Available from: https://www.moj.go.jp/isa/content/001407633.pdf
4. Morita N, Kanamori M, Nochi M, Kondo N. Characteristics of foreign residents in Japan facing difficulties in accessing medical care: A mixed-method study on barriers and effective support measures. (in Japanese) Journal of International Health. 2021;36(3):107-21.
5. Yamaguchi T. Factors affecting the access and use of medical and health Information by foreign residents in Japan: A review of the relevant literature (in Japanese). Japanese Journal of Nursing and Health Sciences. 2023;21:29-40.
6. Takashi S. Challenges of foreign workers and the social security system in Japan. (in

Japanese) Japan Labor Review. 2022;64(7):55-65.

7. Nutbeam D. Health literacy as a public health goal: a challenge for contemporary health education and communication strategies into the 21st century. Health promotion international. 2000;15(3):259-67.

8. Nutbeam D. The evolving concept of health literacy. Soc Sci Med. 2008;67(12):2072-8.

9. Berkman ND, Sheridan SL, Donahue KE, Halpern DJ, Crotty K. Low health literacy and health outcomes: an updated systematic review. Annals of internal medicine. 2011;155(2):97-107.

10. De Wit L, Fenenga C, Giammarchi C, Di Furia L, Hutter I, de Winter A, et al. Community-based initiatives improving critical health literacy: a systematic review and meta-synthesis of qualitative evidence. BMC public health. 2018;18:1-11.

11. McKinn S, Linh DT, Foster K, McCaffery K. Distributed health literacy in the maternal health context in Vietnam. HLRP: Health Literacy Research and Practice. 2019;3(1):e31-e42.

12. Duong TV, Aringazina A, Kayupova G, Nurjanah f, Pham TV, Pham KM, et al. Development and validation of a new short-form health literacy instrument (HLS-SF12) for the general public in six Asian countries. HLRP: Health Literacy Research and Practice. 2019;3(2):e91-e102.

13. Thao NTH, Thanh PH, Tai TP, Hang NT, Nga NT, Linh TTT, et al. Reliability and validity of health literacy questionnaire (new Vietnamese version of HLS-EU-Q47) among mothers of children under 3-year at two vaccination centers in Hanoi in 2019. J Med Res. 2020;127.E6:3.

14. Paudel P, Kovai V, Burnett A, Naduvilath T, Ho SM, Fricke T, et al. Effects of a community-based health education intervention on eye health literacy of adults in Vietnam. International Journal of Health Promotion and Education. 2022;60(3):149-63.

15. Paudel P, Naduvilath T, Kovai V, Phuong HT, Ho SM, & Wilson D. Health literacy of adult Vietnamese population in relation to common eye conditions and factors for not seeking an eye examination. Eye Sci. 2016;31(3):130-39.

16. Dessauvagie A, Dang H-M, Truong T, Nguyen T, Nguyen BH, Cao H, et al. Mental Health Literacy of University Students in Vietnam and Cambodia. International Journal of Mental Health Promotion. 2022;24(3).

17. Tat TN, Tat DN. The associations between mental health literacy and HIV related treatment outcomes among HIV/AIDS outpatients in Vietnam. *TIJPH*. 2022; 20220460517;10(2):90-97.

18. Nguyen LHT, Vo MTH, Tran LTM, Dadaczynski K, Okan O, Murray L, et al. Digital health literacy about COVID-19 as a factor mediating the association between the importance of

online information search and subjective well-being among university students in Vietnam. Frontiers in Digital Health. 2021;3:739476.

19. Cao Ba K, Kaewkungwal J, Pacheun O, Nguyen Thi To U, Lawpoolsri S. Health literacy toward zoonotic diseases among livestock farmers in Vietnam. Environmental health insights. 2020;14:1178630220932540.

20. Cuc DTK, Methakanjanasak N, Trang HTT. Relationships between symptom control, medication management, and health literacy of patients with asthma in Vietnam. Belitung nursing journal. 2021;7(2):131.

21. Stanzel KA, Hammarberg K, Nguyen T, Fisher J. 'They should come forward with the information': menopause-related health literacy and health care experiences among Vietnamese-born women in Melbourne, Australia. Ethnicity & health. 2022;27(3):601-16.

22. Xiao Z, Lee J, Liu W. Korean and Vietnamese immigrants are not the same: Health literacy, health status, and quality of life. Journal of Human Behavior in the Social Environment. 2020;30(6):711-29.

23. Sørensen K, Pelikan JM, Röthlin F, Ganahl K, Slonska Z, Doyle G, et al. Health literacy in Europe: comparative results of the European health literacy survey (HLS-EU). The European journal of public health. 2015;25(6):1053-8.

24. Tsubakita T, Kawazoe N, Ichikawa M, Matsumoto S, Sugawara M. Assessing knowledge-

based and perceived health literacy among Japanese adolescents: a cross-sectional study. Global Pediatric Health. 2020;7:2333794X20944311.

25. Hoang VM, Le TV, Chu TTQ, Duong MD, Thanh NM, Tac Pham V, et al. Prevalence of autism spectrum disorders and their relation to selected socio-demographic factors among children aged 18–30 months in northern Vietnam, 2017. International journal of mental health systems. 2019;13(1):29-37.
