# Supplementary material for: Health literacy assessment and healthcare access difficulties of Vietnamese migrants in Japan: A cross-sectional study
Source: PLoS One. 2026 Mar 20;21(3):e0344665. doi: 10.1371/journal.pone.0344665 (PMC13004346; doi:10.1371/journal.pone.0344665)
Supplement: S2 File — (DOCX) [file pone.0344665.s002.docx]

**Response to the Academic Editor and Reviewers**

We sincerely thank the Academic Editor and three reviewers for their careful review and constructive comments on our manuscript. We greatly appreciate their insightful suggestions, which have substantially helped us improve the clarity, transparency, and overall quality of the manuscript. We have revised the manuscript accordingly and addressed all comments point by point below. In addition, all comments provided in the attached files by Reviewer 1, Reviewer 2 and Reviewer 3 have been carefully reviewed and incorporated into the revised manuscript.　All revisions are highlighted in the revised manuscript using track changes, and a clean version of the revised manuscript has also been provided. We sincerely thank you.

**Response to the Academic Editor**

**Comment:**
Address all comments by reviewers, especially those on the attached documents from Reviewer 1 and Reviewer 3.

**Response:**
We thank the Academic Editor for this guidance. We have carefully reviewed and addressed all comments from all three reviewers, including the detailed comments provided in the attached documents from Reviewer 1 and Reviewer 3. All revisions have been incorporated into the manuscript and are highlighted in the revised version with track changes.

**Response to Reviewer #1**

**Comment 1 on Abstract section:**

Utilization cannot be used interchangebly with access.

**Response:**
We replaced the word “utilization” by “access” and combined these two words into “access” in this manuscript.

This revision can be found in **Abstract (Lines 3).**

**Comment 2 on Introduction section:**

How is this linked to discussions above, because you are now bringing a Vietnam context in Japan. Are you suggesting that same community dynamics are applicable in Japan?

**Response:**

We are suggesting that no community dynamics may be one of reasons of Vietnamese difficulties of poor access to healthcare in Japan.
This response is related to **Introduction (Lines 43).**

**Comment 3 on Introduction section:**

Similar to this statement, I do not see how it builds to your discussion above. My understanding is that their health literacy might be relatively okay in Vietnam compared to Japan, for same reasons you stated above such as language and so forth. How does this assist in building your argument? Or, what is the status quo in Japan?

**Response:**

We intend in this sentence that health literacy research has already employed in Vietnam but not in Japan, where many Vietnamese migrants are living. Therefore, we employed their health literacy research in Japan.

This response is related to **Introduction (Lines 43–45).**

**Comment 4 on Introduction section:**

and?

**Response:**

We are very sorry that we deleted this sentence, because meaning of the sentence was not related with a need of our survey in Japan.
This revision can be found in **Introduction (Lines 46).**

**Comment 5 on Introduction section:**

A statement prior (line 49 to 51) suggest that "generalisation" has aleady been made regarding health literacy outside Vietnam, of which Japan is outside Vietnam too. Why specific interest in Vietnamese in Japan?

**Response:**

Because its generalization covers Australia and United States, not covering Asian countries such as Japan. Therefore, we think that our research on healthcare access difficulties of Vietnamese migrants in Japan is necessary.

This response is related to **Introduction (Lines 48–49).**

**Comment 6 on Objectives section:**

Aim?

**Response:**
We replaced the original sentence by “This study quantitatively assessed health literacy among Vietnamese migrants in Japan and examined their health access as well as the difficulties they encounter.” to make the objectives clear.

This revision can be found in **Objectives (Lines 51).**

**Comment 7 on Objectives section:**

Is your interteres "utilization" or "access". The two means two different things.

**Response:**

We replaced the word “utilization” by “access” through this manuscript.
This revision can be found in **Objectives (Lines 53).**

**Comment 8 on Materials and methods section:**

Which prior studies? Please sustantiate as to how are these specific migrants likely to face challenges with health literacy.

**Response:**

The prior studies are [14-15], which was added to the manuscript. These migrants in Japan are likely to face challenges in low health literacy, because the studies indicated that language and cultural barriers continued to restrict access to health information outside Vietnam.
This revision can be found in **Materials and methods (Lines 59).**

**Comment 9 on Materials and methods section:**

Duration of stay usually has a certain influence in studies like this, was that not considered. If so, what was the duration, and if not, why not.

**Response:**

The duration of their stay is shown in Table 2, where more than 80 % of the migrants were staying in Japan for less than 7 years, so that their duration was not so long. Though it is important to take the duration into consideration, we did not consider it in the study, and this is our study’s limitation.

This revision can be found in **Materials and methods (Lines 61-62).**

**Comment 10 on Materials and methods section:**

This contradicts a statement provided earlier on ethics statement, where you indicated that consent was regarded by virtue of filling in the questionnaire.

**Response:**
In the first part of our questionnaire, ethical explanations were written. We asked participants to read them before answering the questionnaire and to answer it only if they got informed consent. We replaced the original explanation of informed consent in the Ethical statement by more detailed explanation.

This response is related to **Materials and methods (Lines 64–66).**

**Comment 11 on Materials and methods section:**

Was it used in this study too?

**Response:**

Yes, we used the HLS-SF12 tool in our research. Table 6 shows the result of its measurement.
This response is related to **Materials and methods (Lines 76).**

**Comment 12 on Materials and methods section:**

You are explaining what are these tools used for, but not clarifying if they were used in this study or not, or which one was used and which was not. Please clarify.

**Response:**

We used the HLS-SF12 as a self-reported tool and the funHLS as a test-based tool. Table 6 shows the result based on the former tool and Table 7 shows the result based on the latter one.
This response is related to **Materials and methods (Lines 80-81).**

**Comment 13 on Results section:**

I think you should add context here, is it trust in quality for Japanese or Vietnamese medication?

**Response:**
We added the words “of Japanese medication” into the sentence.

This revision can be found in **Results (Lines 149).**

**Comment 14 on Results section:**

What does it mean when the mean score is 9.12, the significance thereof? Based on the maximum possible score.

**Response:**
The tool, HLS-SF12, comprises of 3 domains, each of which has four choices, 1-4, such as 1-4 score, respectively. Therefore, each participant has a total score between 3 and 12 in each domain of HLS-SF12 tool. The mean score (M) 9.12 indicates that the mean of participants total scores in the healthcare domain was 9.12, relatively high and close to maximum 12.

This response is related to **Results (Lines 184).**

**Comment 15 on Results section:**

Refer to a question earlier on maximum possible score and reporting on the significance of the mean value.

**Response:**

The tool, HLS-SF12, comprises of 3 domains, one of which is the disease preventive. The mean score (M) for women was 10.26, close to the maximum 12, and higher than the score for men 9.62. Women were likely to self-report their health literacy more positive than men in the data.
This response is related to **Results (Lines 187–189).**

**Comment 16 on Results section:**

refer to previous question on mean valu**e.**

**Response:**
For the health promotion domain, the mean score (M) was 10.70 for women and 10.76 for men. There are no sex differences in their mean value.

This response is related to **Results (Lines 193).**

**Comment 17 on Results section:**

Were men expected to score on amenorrhea?

**Response:**

No, they were not. Men were not expected to score highly on amenorrhea, though even men are preferable to have such a knowledge.
This response is related to **Results (Lines 208).**

**Comment 18 on Discussion section:**

Please quantify, what is many? or how many is many.

**Response:**

Among the 137 participants, 77 (56.2%) reported seeking medical care for illnesses or injuries since arriving in Japan

This response is related to **Discussion (Lines 225–226).**

**Comment 19 on Discussion section:**

number? or percentage?

**Response:**
Table 3 shows their difficulties in doctor visits.

This response is related to **Discussion (Lines 228–229).**

**Response to Reviewer #2**

**Comment 1 on Abstract section:**

The manuscript is well written and data well presented. However, being the first study of its kind, the authors may rephrase their first sentence in their abstract to read. "their healthy literacy as a key concern for ensuring equitable access to healthcare is unknown."

**Response:**
We replaced the original sentence by “their healthy literacy as a key concern for ensuring equitable access to healthcare is unknown” to make the focus of the study clear. Thank you for your precise advice.

This revision can be found in **Abstract (Lines 2–3).**

**Comment 2 on Materials and methods section:**

Further, under the section named Data analysis, the authors have stated the software used and the descriptive analyses, however, they need to specify the tests and model (s) used in the study.

**Response:**

We added the sentence "A goodness-of-fit test was used to compare men and women in Table 7”.
This revision can be found in **Materials and methods (Lines 90).**

**Response to Reviewer #3**

**Comment 1 on Ethics Statement:**

The consent procedure ("consent... considered to be given by the act of completing the questionnaire") should be explicitly labeled as implied consent.

**Response:**

We labeled the words “implied consent” in Ethics statement.
This revision can be found in **Ethics Statement.**

**Comment 2 on Abstract section:**

convenience sampling greatly hinders generalization of findings.

**Response:**
Yes, convenience sampling greatly hinders generalization of findings. We think that convenience sampling is a limitation of our study.

This response is related to **Abstract (Lines 5)**.

**Comment 3 on Abstract section:**

Consider specifying direction of gender disparity (e.g., "with women/men scoring lower in...") to make the finding more immediate.

**Response:**

We added the words” where women were likely to score higher than men” in the sentence.
This revision can be found in **Abstract (Lines 9)**.

**Comment 4 on Abstract section:**

A stronger conclusion that calls for action would be better and offer direction towards better outcomes.

**Response:**
We deleted the words “We also hope that“, and made the conclusion a stronger expression than before.

This revision can be found in **Abstract (Lines 13–14)**.

**Comment 5 on Materials and methods section:**

Please clarify the timeline. are the dates accurate?

Clarify the recruitment window (28/07/2024 to 16/01/2025 seems unusually long or is a typo for 2023-2024?).

**Response:**
We replaced the date expression from 28/07/2024, 16/01/2025 to July 28, 2024, January 16, 2025, respectively. Participants recruitment duration was 6 months. Because we asked Vietnamese support organizations across Japan to disseminate a web-based survey through their related social media platforms, 6 months were needed to collect our participants data.

This revision can be found in **Materials and methods (Lines 67)**.

**Comment 6 on Results section:**

These numbers don't add up to 77 could this mean that the total number was higher than 77.

**Response:**
We added the words “multiple answers” in the title of Table 3, which was calculated based on multiple answers, so that the sum of numbers does not meet 77.

This revision can be found in **Results (Lines 134)**.

**Comment 7 on References:**

Formatting is inconsistent (some titles capitalized, others not). Check journal style

**Response:**
We corrected formatting inconsistency of capital letters in the References according to the journal style.

This revision can be found in **References (Lines 322–374)**.
